# The Proteome Landscape of *Giardia lamblia* Encystation

**DOI:** 10.1371/journal.pone.0083207

**Published:** 2013-12-31

**Authors:** Carmen Faso, Sylvain Bischof, Adrian B. Hehl

**Affiliations:** 1 Institute of Parasitology, University of Zurich, Zurich, Switzerland; 2 Department of Biology, ETH Zurich, Zurich, Switzerland; Cornell University, United States of America

## Abstract

*Giardia lamblia* is an intestinal protozoan parasite required to survive in the environment in order to be transmitted to a new host. To ensure parasite survival, flagellated trophozoites colonizing the small intestine differentiate into non-motile environmentally-resistant cysts which are then shed in the environment. This cell differentiation process called encystation is characterized by significant morphological remodeling which includes secretion of large amounts of cyst wall material. Although much is known about the transcriptional regulation of encystation and the synthesis and trafficking of cyst wall material, the investigation of global changes in protein content and abundance during *G. lamblia* encystation is still unaddressed.

In this study, we report on the quantitative analysis of the *G. lamblia* proteome during encystation using tandem mass spectrometry. Quantification of more than 1000 proteins revealed major changes in protein abundance in early, mid and late encystation, notably in constitutive secretory protein trafficking. Early stages of encystation were marked by a striking decrease of endoplasmic reticulum-targeted variant-specific surface proteins and significant increases in cytoskeleton regulatory components, NEK protein kinases and proteins involved in protein folding and glycolysis. This was in stark contrast to cells in the later stages of encystation which presented a surprisingly similar proteome composition to non-encysting trophozoites. Altogether these data constitute the first quantitative atlas of the Giardia proteome covering the whole process of encystation and point towards an important role for post-transcriptional control of gene expression in Giardia differentiation. Furthermore, our data provide a valuable resource for the community-based annotation effort of the *G. lamblia* genome, where almost 70% of all predicted gene models remains “hypothetical”.

## Introduction

Some of the most widespread protozoan parasites rely on the development of an environmentally resistant infectious form (ERIF). ERIFs come in the form of cysts and, more specifically, oocysts when they arise from a sexual stage in the parasite's life cycle. Environmental shedding of mature ERIFs allows for per oral parasite transmission to a new host, thus achieving completion of the infectious cycle.

Similarly to species such as *Toxoplasma gondii* [1], *Eimeria tenella* [2], *Entamoeba invadens* [3] and most recently *Dientamoeba fragilis* [4], the diplomonad *Giardia lamblia* (syn. *G. duodenalis*, *G. intestinalis*) requires the formation of an environmentally-resistant cyst for transmission to a new host [5]. Giardial cysts are shed in fecal matter which may contaminate water sources. Following ingestion, they differentiate into flagellated excyzoites after passage through the stomach. These intermediate cell stages rapidly undergo 2 rounds of cell division, giving rise to 4 fully developed trophozoites. This infection usually develops into a full-fledged parasitic disease known as giardiasis which accounts for the majority of non-bacterial diarrheal waterborne illness [6].

Morphologically, encystation of giardial trophozoites is a striking form of cell differentiation during which a flagellated pear-shaped binucleate trophozoite becomes an oval tetranucleate cyst. Recent work has contributed to the unraveling of key aspects in the initiation and progression of encystation [7]–[9], including the detailed characterization of encystation specific vesicle (ESV) neogenesis [10]. These organelles are deputed to the accumulation and maturation of stage-specific cyst wall material (CWM) composed of at least 3 cyst wall proteins (CWP1-3) complexed with β(1-3)-N-acetyl-d-galactosamine (GalNAc) polymer [11]. CWM is eventually deposited in juxtaposition to the plasma membrane. ESVs possess several Golgi-like features such as their dependence on active endoplasmic reticulum (ER) exit sites [10], their association to known Golgi-specific protein trafficking components [12], [13], their sensitivity to brefeldin A [14] and their ability to delay, chemically modify and partition cyst wall cargo during secretory transport [15]. Trafficking of mature CWM from ESVs to the cell surface, where it forms the cyst wall is tightly regulated and rapid, occurring alongside profound changes in the morphology of the trophozoite 20–24 h after induction of encystation. During cyst formation, trophozoites appear progressively rounded; major cytoskeletal components such as the flagella, the adhesive disk and the median body are almost entirely disassembled [16] while other subcellular compartments such as the ER are profoundly re-organized (Faso and Hehl, unpublished material). This process is accompanied by 1 and 2 rounds of nuclear and DNA replication, respectively, yielding a cyst with 16N ploidy and 4 nuclei [17], [18].

We hypothesized that the proteome of Giardia trophozoites progressing through distinct stages of encystation [15] is marked by the differential regulation of specific metabolic pathways and structurally-important proteins. Comparative microarray analysis revealed only 18 up-regulated and 10 down-regulated genes during the first 7 hours of encystation, suggesting that differentiating parasites experience only minor changes in their transcriptome [7]. In addition, a transcriptome study using serial analysis of gene expression (SAGE) of the Giardia life cycle identified 42 genes as encystation markers [19]. Further information on the Giardia trophozoite transcriptome was recently provided by two studies using microarray and RNA-seq methodologies which also uncover lineage-specific genome-wide expression differences in four genetically distinct Giardia genotypes [20], [21]. Complementing transcript studies, mass spectrometry (MS)-based proteomics has successfully been used not only to investigate metabolic changes occurring at defined stages of encystation but also to dissect intracellular protein localization in mitosomes and the basal body [22]–[24].

In this study, we report on the large-scale quantitative analysis of the *G. lamblia* proteome covering the whole process of encystation, from CWP accumulation at the ER through to selective condensation in mature ESVs. We applied label-free shotgun proteomics [25], [26] and quantified more than 1000 proteins in non-encysting trophozoites and cells induced to encyst *in vitro* over a 14 hour time period. This work serves multiple purposes: It sheds light on the strong regulation of protein abundance that characterizes early stages of encystation, providing evidence for a re-organization of intracellular trafficking routes and cytoskeletal components. Furthermore, it effectively complements existing data on the transcriptional regulation of Giardia encystation [7], [19], thus providing a more complete picture of this key cell differentiation process. Finally, this quantitative data provides a valuable resource to all future encystation-related investigations and contributes significantly to ongoing community-based annotation efforts of the *G. lamblia* genome.

## Results and Discussion

### Proteome overview from trophozoites to encysted cells

**Figure 1:**
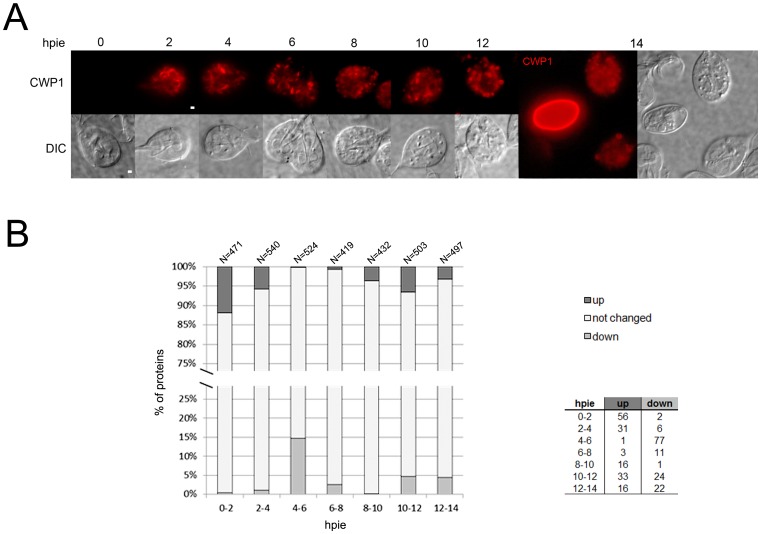
Proteome profile of *G. lamblia* trophozoites analyzed at 0, 2, 4, 6, 8, 10, 12 and 14hpie. (A) Upper row: Wide-field immunofluorescence microscopy analysis of CWP1 (red) localization in representative *G. lamblia* trophozoites induced to encyst over a 14 hr time period using the 2-step encystation method [29]. Between 2 and 4hpie, CWP1 is mainly localized to the ER. From 6 to 8hpie, ESVs emerge and develop, reaching the partitioning phase for CWPs between 10 and 12hpie. At 14hpie, cyst production is already under way within a population of late-encysting cells. Lower row and far right: corresponding bright-field images. Condensed-core ESVs become visible at 8hpie (white arrow). hpie: hours post induction of encystation. Scale bars: 1 µm. (B) Regulation of protein abundance within 2-hour transitions during the 14 hour encystation time-course experiment. Based on relative quantitative information by nSpC for each identified protein (further information in Table S1), protein abundance across each transition was either up-regulated (up), down-regulated (down) or did not change. The total number of proteins for each dataset is indicated above each bar. Transitions between 0-2-4 and 4-6-8hpie showed a trend for increased and decreased protein abundances respectively while the last 4 hours of encystation were marked by a slight tendency of increased abundances. The associated table reports the exact number of proteins in each category. hpie: hours post induction of encystation.

To obtain first insights into the complexity of the Giardia proteome and its dynamics during encystation, we performed a single 2 hour-sampling time course. As a method for the induction of encystation, we chose the 2-step induction protocol [27] because, in contrast to the commonly-used high-bile method [28], it is not selective and allows for a more reliable and reproducible timing of CWM biosynthesis and ESV development [29]. Furthermore, the 2-step method was shown to be equally effective as the lipid-depletion induction approach for the regulation of the core set of encystation genes [7]. Cultured Giardia trophozoites (*ca.* 40 million cells/time-point) were either grown and harvested in standard TYI-S-33 culture medium (non-induced 0hpie control) or first grown in pre-encysting medium for 44 hours, moved to encysting medium and then harvested at 2 hr intervals, for 14 hours. To ensure correct induction of CWM biosynthesis and trafficking to ESVs, we used immunofluorescence assays to monitor CWP1 accumulation and translocation in aliquots of non-encysting and encysting cells harvested during the time-course; CWP1 is commonly used as a marker for CWM trafficking and ESV neogenesis [27]. Wide-field microscopy observation of fixed cells labeled with anti-CWP1 monoclonal antibody conjugated to the Texas-Red fluorophore demonstrated the timely induction of CWP1 expression in 70–80% of the cells (Figure 1A) and the progressive development of ESVs during their previously documented stages of neogenesis and maturation [15]. Representative single cell examples of populations harvested at given time-points are shown in Figure 1A. Although the 14hpie time-point presented occasional cysts (Figure 1A, far right), these were discarded and only attached cells were harvested.

Having confirmed the correct induction of encystation, harvested cells were lysed in a SDS-containing buffer and proteins were quantified using the biconchicinic acid (BCA) method. Similar protein concentrations (∼10 µg/µl) were obtained for all samples and 40 µg of total protein/sample were resolved by SDS-PAGE (Figure S1A). In-gel tryptic digestion was performed and resulting peptides were measured by tandem mass spectrometry (MS) using a high mass accuracy Orbitrap mass spectrometer. Following database searches, we identified a total of 688 proteins with at least two unique peptides (Table S1). Single hits were discarded. Methods and criteria used for MS, protein identification, and quantification are described in detail below. The number of proteins identified at each time-point and the overlap between these was comparable, suggesting that the Giardia proteome remains overall robust during encystation (Table S1 and Figure S1B).

To test whether our experimental approach could reliably detect changes in the proteome of encysting cells, we searched our protein datasets for known markers of encystation [7], [30]. We detected a time-dependent increase in the total number of identified peptides for several proteins (Table 1) including CWP2 (GL50803_5435) and 5 enzymes belonging to the encystation-regulated GalNAc biosynthesis pathway [30]: glucose-6-phosphate isomerase (GL50803_9115), phosphoacetylglucosamine mutase (GL50803_16069), UDP-glucose-4-epimerase (GL50803_7982), UDP-N-acetylglucosamine pyrophosphorylase (GL50803_16217) and glucosamine-6-phosphate deaminase (GL50803_8245), consistent with their induction by encystation [30]. Furthermore, transcript levels for open reading frames (ORF) GL50803_ 5435, 9115, 16069 and 8245 were previously shown to be significantly up-regulated by at least 2 fold in trophozoites induced to encyst using the 2-step method (protocol A in [7]); ORF GL50803_7982 and GL50803_16217 transcripts were also increased, albeit at less than 2-fold. In addition, we identified 10 of the 42 proteins that were shown to have increased transcript levels during encystation induced by high bile concentrations [19]. The number of peptides identified for 8 out of these 10 increased during the 14 hour time-course (Table 1). We also identified 11 putative encystation markers that were previously defined in a two-dimensional polyacrylamide gel electrophoresis (2D-PAGE) -based analysis comparing the proteome of non-encysting trophozoites to cysts (Table 1; Protein up 2D PAGE) [22]. We observed a modest increase for these previously defined encystation markers in the later time-points of encystation. Overall, our data is consistent with previous reports, thus validating our experimental strategy and indicating that it is suitable to detect changes in the proteome of encysting Giardia cells.

**Table 1:** Encystation markers identified at 0, 2, 4, 6, 8, 10, 12 and 14hpie.

		Total number of peptides								Protein up	Transcript up	
ORF	Annotation	0 hpie	2 hpie	4 hpie	6 hpie	8 hpie	10 hpie	12 hpie	14 hpie	2D PAGE	Microarray	SAGE
**GL50803_112103**	Arginine deiminase	93	98	104	86	84	102	123	115	Y	-	-
**GL50803_17063**	Pyruvate-flavodoxin oxidoreductase	89	54	96	74	84	96	94	88	Y	-	-
**GL50803_103676**	Alpha-tubulin	85	74	86	89	96	94	117	117	Y	-	-
**GL50803_10311**	Ornithine carbamoyltransferase	77	53	65	70	74	88	104	121	Y	-	-
**GL50803_88765**	Cytosolic HSP70	76	82	82	91	87	99	106	118	y	-	-
**GL50803_98054**	Heat shock protein HSP 90-alpha	37	28	36	40	36	40	41	46	y	-	-
**GL50803_15409**	Kinase, NEK	34	28	34	30	35	36	38	42	y	-	-
**GL50803_12102**	Elongation factor 1-gamma	31	30	32	28	37	35	33	42	y	-	-
**GL50803_7532**	Vacuolar ATP synthase catalytic subunit A	21	21	22	22	19	22	21	16	y	-	-
**GL50803_4812**	Beta-giardin	18	24	23	26	28	26	45	39	y	-	-
**GL50803_9115**	Glucose-6-phosphate isomerase	11	12	11	16	20	23	27	25	-	y	-
**GL50803_4197**	Phosphatidylinositol transfer protein alpha isoform	7	8	7	9	8	7	11	12	-	-	y
**GL50803_6184**	Branched-chain amino acid aminotransferase lateral transfer candidate	3	2	7	3	5	4	4	3	y	-	-
**GL50803_102813**	Protein 21.1	-	6	5	4	5	4	5	8	-	y	-
**GL50803_5435**	Cyst wall protein 2	-	2	3	7	20	21	25	26	-	y	y
**GL50803_8172**	Dynein heavy chain	-	2	3	2	3	3	2	3	-	-	y
**GL50803_8245**	Glucosamine-6-phosphate deaminase	-	-	-	7	7	13	39	35	-	-	y
**GL50803_16069**	Phosphoacetylglucosamine mutase	-	-	-	-	3	5	5	4	-	y	y
**GL50803_40376**	High cysteine non-variant cyst protein	-	-	-	-	2	-	2	2	-	-	y
**GL50803_7982**	UDP-glucose 4-epimerase	-		-	-	-	6	11	7	-	-	y
**GL50803_2897**	Furin precursor putative serine protease	-	-	-	-	-	2	4	5	-	-	y
**GL50803_16424**	Hypothetical protein	-	-	-	-	-	-	4	3	-	-	y
**GL50803_16217**	UDP-N-acetylglucosamine pyrophosphorylase	-	-	-	-	-	-	2	6	-	-	-
**GL50803_14626**	Oxidoreductase, short chain dehydrogenase	-	-	-	-	-	-	2	2	-	y	-
**GL50803_8377**	Hypothetical protein	-	-	-	-	-	-	2	-	-	-	y

Total number of identified peptides for 25 known encystation protein markers over the 14 hour encystation time-course experiment. CWP2 (GL50803_5435) and members of the GalNAc biosynthesis pathway (ORFs GL50803_ 9115, 16069, 7982, 16217 and 8245) are highlighted in orange. A “y” for “yes” marks ORFs previously reported to be induced by encystation in 2D PAGE [22], microarray [7] or SAGE experiments [19]. hpie: hours post induction of encystation.

We next obtained relative quantitative information for each identified protein by using normalized spectral counting (nSpC) [25], [26], [31] (Table S1). Differentially abundant proteins between two time-points were required to have a minimum twofold change in abundance and to have been identified with at least 5 peptides in one of the two time-points (Table S1). Proteins detected in only one time point were also required to have at least 5 identified peptides to be considered significantly more abundant. In the 14 hour encystation time-course, we observed that the abundance of many proteins increased during the first 4 hours post induction of encystation (hpie) (Figure 1B). In contrast, the transitions between 4–6 and 6–8hpie showed a trend for decreased protein abundances while the last 4 hours of encystation (8–12hpie) were marked by a slight tendency of increased abundances. This suggests that, in our experimental set-up, major changes in the proteome of encysting cells appear to take place at the time-points 0, 4, 8 and 12hpie. Furthermore, when the 2-step encystation method is employed, these time-points correlate with well-defined stages of CWM trafficking and ESV neogenesis [15]. At 4hpie, emerging ESVs become recognizable. Progression to 8hpie is required for these organelles to transition from an accumulating to a partitioning phase for CWPs 1–3 deposition [15], including maturation of CWP2 by proteolytic cleavage [15], [32]. Furthermore, induction to 12hpie is characterized by the sorting and secretion of CWM to the outer layer of the nascent cyst wall. We therefore selected these time-points for in-depth characterization.

### In-depth analysis of the Giardia proteome during encystation

To quantitatively assess the changes observed during the 14 hour time course, we profiled the proteome of Giardia cells after 4, 8 and 12hpie in 3 biological replicates. We grew, encysted, harvested and lysed cells and performed tandem MS as described previously for each selected time-point of encystation in completely separate experiments.

**Figure 2:**
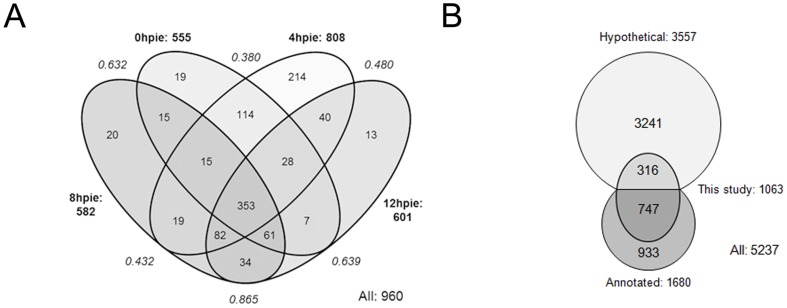
Venn diagrams depicting proteome profiles of *G. lamblia* trophozoites analyzed at 0, 4, 8 and 12hpie. (A) The proteomic analysis done in triplicates of *G. lamblia* trophozoites after 4, 8 and 12hpie yielded a total of 960 proteins with at least two unique peptides (further information in Table S2). Although the total number of proteins identified in each of the 4 time-points (in bold) was comparable, mean Spearman rank correlation coefficients (in italics) indicated that the 4hpie time-point differed the most with respect to the other 3 time-points. hpie: hours post induction of encystation. (B) In this study, we identified a total of 1063 proteins, 316 of which were annotated as “hypothetical” in the Giardia Genome Database. Based on its current release (GiardiaDB 3.0; 11^th^ of March 2013), the *G. lamblia* assemblage A WB strain genome was assigned 5237 protein-encoding genes, with 3557 genes awaiting annotation.

In total, 960 proteins were detected with at least two unique peptides (Figure 2A, Table S2). 585 proteins were identified both in the 14 hour time course and the triplicate experiment while 103 and 375 were unique to the two datasets respectively. Altogether, 1063 proteins were identified providing the first large-scale overview of the Giardia proteome. All datasets were deposited in the PRIDE database (www.ebi.ac.uk/pride) [33] and are accessible under the experiment numbers 26860–26879. 316 of the 1063 identified proteins were annotated as “hypothetical” in the Giardia Genome Database (GGD; [34]) and none encoded by “deprecated” ORFs (Figure 2B). Based on its current release (GiardiaDB 3.0; 11^th^ of March 2013), the *G. lamblia* assemblage A WB strain genome [35] is predicted to contain a total of 5901 protein-encoding genes, although only 5237 have been assigned gene identification numbers. 3557 of these genes are annotated as coding for “hypothetical” proteins. This annotation is maintained by the database curators for predicted proteins whose putative function/homology has not (yet) been assigned. Comparison of the abundances of annotated and “hypothetical” proteins at 0hpie indicated that annotated proteins were overall more abundant than “hypothetical” proteins (Figure S1C). This is consistent with previously published RNA-seq data from different *G. lamblia* assemblages (Figure S1C) [21].

**Figure 3:**
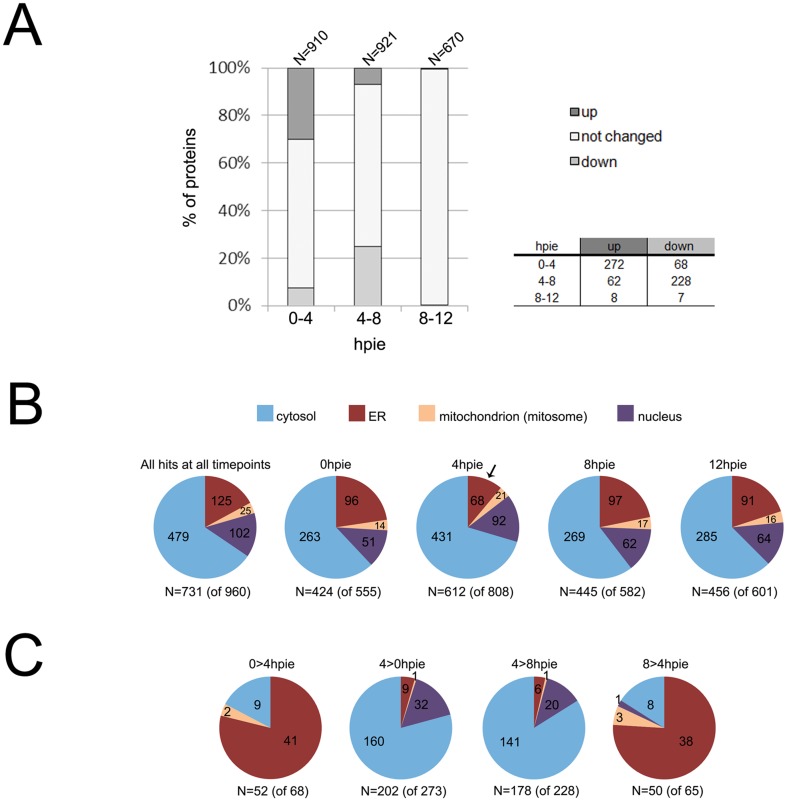
Protein abundance and predicted subcellular distribution of *G. lamblia* trophozoites analyzed at 0, 4, 8 and 12hpie. (A) Comparison of changes in protein abundance between 0 and 4hpie (0–4), 4 and 8hpie (4–8) and 8 and 12hpie (8–12). Based on relative quantitative information by nSpC for each identified protein (further information in Table S2), protein abundance across each 4 hour interval was either up-regulated (up), down-regulated (down) or did not change. The total number of proteins for each dataset is indicated above each bar. In total, the abundance of 342 and 303 proteins was significantly increased or decreased between two different time-points. The 4hpie time-point presented the highest number of both detected and significantly regulated proteins, while only few changes were recorded at 12hpie compared to 8hpie. The associated table reports the exact number of proteins in each category. hpie: hours post induction of encystation. (B) Target P and NucPred predictions for the subcellular distribution of all proteins detected at each time-point. The predicted contribution of mitochondrion (mitosome), nucleus and cytosol localized proteins was similar across time-points. In contrast, the number of proteins predicted to traffic to or through the ER was reduced by *ca.* 50% at the 4hpie time-point (black arrow). The number of proteins which satisfied both algorithm thresholds for reliability is indicated beneath the respective pie-chart; in brackets, the overall number of proteins for each dataset is indicated. hpie: hours post induction of encystation. (C) Target P and NucPred predictions for the subcellular distribution of significantly regulated proteins between 0 and 4 and 4 and 8hpie. Proteins more abundant at 4hpie compared to 0 and 8hpie (4>0hpie and 4>8hpie) were significantly depleted for predicted ER-targeted hits and enriched for putative nuclear targeted proteins. This view was almost reversed for proteins with higher abundance at 0 and 8hpie compared to 4hpie (0>4hpie and 8>4hpie). The number of proteins which satisfied both algorithm thresholds for reliability is indicated beneath the respective pie-chart; in brackets, the overall number of proteins for each dataset is indicated. hpie: hours post induction of encystation.

The total number of proteins identified in each of the 4 time-points was comparable and 353 proteins overlapped between all datasets, indicating good reproducibility of the chosen experimental approach (Figure 2A). As also observed in the 14 hour time course, the comparable number of proteins identified at each time-point suggested that the Giardia proteome remains overall robust during encystation (Table S1 and Figure S1B). In support of this view, a previous comparative microarray analysis revealed only 28 differentially regulated genes during the first 7 hours of encystation [7]. Mean Spearman rank correlation coefficients indicated that the 4hpie differed the most with respect to the other 3 time-points (Figure 2A, numbers in italics). This is consistent with the 14 hour time-course experiment and suggests that most of the changes in protein abundance during in Giardia encystation occur early in the process. We next used the quantitative information obtained using nSpC in each biological triplicate to define proteins that were significantly more abundant in each time-point (Table S2). These proteins have a minimum twofold change in abundance (mean nSpC), a P value <0.05, and were identified with at least 5 peptides in one biological replicate. Proteins detected in a single time-point were also required to have at least 5 identified peptides to be considered significantly more abundant. In total, the abundance of 342 and 303 proteins, respectively, was significantly increased or decreased between two different time-points (Figure 3A).

In our experimental conditions, the 4hpie time-point presented the highest number of both detected and significantly regulated proteins, while only few changes were recorded at 12hpie compared to 8hpie (Figure 3A). This was consistently shown in all 3 biological replicates (Table S2) and in the 14 hour time-course experiment (Figure 1B). The analysis of the 100 most abundant proteins identified at 0, 4, 8 and 12hpie showed that their abundances vary between the 4 different time points, especially at 4 hpie (Figure S2). Variations in the quantity of highly abundant proteins in a complex extract can impact the detection by mass-spectrometry of low abundance proteins, possibly leading to an increased detection at 4hpie. Altogether, these data show how the early stages of encystation present the strongest degree of regulation at protein level. Furthermore, we and others (Staffan Svard, personal communication) have systematically observed how Giardia cultures progressing through encystation show lower counts for attached trophozoites at *ca.* 4hpie compared to non-encysting cultures. Interestingly, *G. lamblia* was shown to differentiate in the G_2_ stage of the cell cycle and encystation frequency was shown to depend on the number of cells that were arrested in G2 [18]. It is therefore possible that cells in early encystation experience a bottleneck in attachment efficiency which depends on the stage of the cell cycle they are in upon induction.

Interestingly, proteins identified at 0 and 8hpie presented close to 80% overlap (Table S2), reaching almost 88% when the same comparison was done between 8hpie and 12hpie. In contrast, only 58% and 56% of all proteins found at 4hpie were also found at 8hpie and at 0hpie, respectively. Altogether, this suggests that in our experimental conditions, 4 hr-encysting trophozoites differed the most with respect to either non-induced cells or to trophozoites in late encystation. Although we detected more proteins at 4hpie compared to the other time-points, this does not interfere with accurate quantification using nSpC. This method takes into account the size of each dataset (i.e. the total number of identified peptides, and proteins) and generates normalized protein abundances separately for each protein in all datasets. Furthermore, aside from proteins which were significantly more abundant at 4hpie compared to 0 and 8hpie (272 and 228 proteins; Figure 3A), we also found 68 and 62 proteins that were significantly more abundant at 0 and 8hpie compared to 4hpie, respectively (Figure 3A). This suggests that the higher number of detected proteins at 4hpie had only a minor impact on the accuracy of protein quantification by nSpC.

### Constitutive secretion is remodeled in early encystation

We next asked whether protein regulation during encystation is accompanied by changes in subcellular protein distribution. Given the compartments present in the giardial cell [36], we applied the widely-used algorithms Target P [37] and NucPred [38] to predict the subcellular distribution of all proteins detected at every time-point (Table S2). In all 4 profiled time-points, subcellular localization distribution showed an overall similar contribution for mitochondria/mitosomes, nucleus and cytosol localized proteins (Figure 3B). However, the number of proteins predicted to traffic to or through the ER at 4hpie was reduced by *ca.* 50% (Figure 3B; black arrow). The decreased representation of predicted secreted proteins was even more pronounced when the subcellular localization analysis was performed for proteins significantly more abundant at one time-point (Figure 3C). We noticed that more than one third of the predicted secreted proteins at 0, 8 and 12hpie were variant-specific surface proteins (VSPs). VSPs are secreted in large numbers to the surface of Giardia trophozoites, forming protein coats hypothesized to play a role in immune evasion and establishment of chronic infection in parasitized hosts [5]. Although only one VSP from a repertoire of ∼200 VSP gene members in the Giardia genome is expressed on the surface of individual Giardia cells at any time, in the absence of immunological selection, cultured populations typically contain a mixture of parasites presenting different VSPs [21]. Antigenic switching to a different type of VSP occurs spontaneously, both in culture and *in vivo* [39] and its regulation appears to depend on post-transcriptional phenomena including an RNAi-related pathway [40] and/or microRNAs [41]. The large number of VSPs suggests this protein family may constitute the bulk of ER-targeted proteins in Giardia trophozoites.

We detected a total of 47 VSPs across all time-points (Table S3). The regulation of transcript levels in encysting trophozoites for 22 of these VSPs is similar to the data we present for the corresponding protein products [7].The 0hpie dataset includes the highest number of detected VSPs with 45 family members, of which 29 presented significant differential expression (Table S3). 36 and 35 VSP family members were identified at 8hpie and 12hpie, respectively. Strikingly, only 13 VSPs were identified in the 4hpie dataset, indicating that the diversity of the repertoire of expressed VSPs in early encysting trophozoites was significantly reduced (Table S3). The reduced VSP variety at 4hpie was consistent with the observed drop in predicted ER-targeted proteins. Except for VSP GL50803_9276, 12 of the 13 VSPs detected at 4hpie were present across all time-points. VSPs GL50803_ 103992, 111933, 113304, 134710, 14331 and 40591, were only detected in non-encysting trophozoites. In contrast to this, VSPs GL50803_137606 and 9276 were only detected in encysting trophozoites. Taken together, this data indicates that induction of encystation affects VSP diversity in trophozoites early during encystation. There are at least two interpretations for the striking bottleneck in VSP diversity uncovered by our data may be interpreted in at least 2 ways. On the one hand, a reduction in detected VSP variants in the population may be due to a loss of trophozoite subpopulations carrying specific VSPs. These populations would lose ground in favor of subpopulations carrying other VSP antigens which, for reasons yet unknown, could be more compatible with survival and proliferation in the media conditions used to induce encystation. In support of this hypothesis, our data records an overall decrease in VSP diversity, from 45 at 0hpie to 36 and 35 at 8 and 12hpie, respectively. VSPs GL50803_ 103992, 111933, 113304, 134710, 14331 and 40591, were only detected in non-encysting trophozoites and, except for ORFs GL50803_111933 and 40591, this is consistent with previously measured transcript levels in the early stages of encystation [7]. On the other hand, out of the 13 VSPs detected throughout the 4 hour time-course, 11 VSPs were significantly more abundant at 0 and 8hpie compared to 4hpie, suggesting that these VSPs may correspond to a core subset of surface antigens whose abundance is specifically down-regulated at 4hpie. This could play a role in accommodating for the imminent trafficking of large amounts of CWM, by reducing constitutive secretion of VSPs and of other surface antigens. In support of this idea, secretory trafficking-related proteins such as the signal recognition particle (SRP 68 kDa; ORF GL50803_8916), COPII component Sec31 (GL50803_2562) and coatomer subunits alpha (GL50803_11953) and gamma (GL50803_5603) were found to be significantly more abundant at 4hpie compared to both 0 and 8hpie (Table S2). Therefore, early encysting cells may promote stage-specific expression and trafficking of CWPs by reducing overall constitutive secretion and increasing abundance of SRP and of other trafficking machinery. Coatomer subunits in other eukaryotes are known to mediate retrograde protein trafficking from the Golgi apparatus to the ER and to be involved in Golgi cisternal maintenance [42]. The absence of a stable Golgi compartment in *G. lamblia*, supplanted by *de novo* generated ESVs which were previously shown to be associated to coatomer [12], raises the question of the presence of retrograde transport routes involving nascent ESVs.

Another class of secretory proteins which also appeared to be significantly less abundant at 4hpie with respect to 0hpie and 8hpie are the poorly-characterized high cysteine membrane proteins (HCMps). We detected a total of 14 up-regulated HCMps at 0hpie and at 8hpie with respect to 4hpie. Except for 3 of these (GL50803_91099, 16716 and 9620), corresponding transcript levels reportedly increased at 3–7hpie compared to a vegetative state, albeit less than 2-fold [7]. Similarly to VSPs, HCMps are rich in cysteine (>10%) and usually contain more than 20 repeats of the CXXC and/or the CXC motif. The latter motif distinguishes them from VSPs, as these very rarely exhibit the CXC motif [43]. Furthermore, HCMPs lack the C-terminal CRGKA epitope which is a hallmark of VSPs. One member of the HCMP family (HCNp; GL50803_40376) was shown to be increasingly expressed at 21 and 42hpie and in water-resistant cysts [44]. Furthermore, HCNp localized to nuclei in non-encysting trophozoites and to ESVs and to the cyst's wall and cell body [44]. It is noteworthy that, similarly to VSPs, more HCMps were differentially abundant at 8hpie than at 0hpie, suggesting a common regulatory mechanism during encystation for constitutively-secreted proteins such as VSPs and HCMps.

### Functional annotation analysis defines clusters of differentially regulated metabolic functions

**Figure 4:**
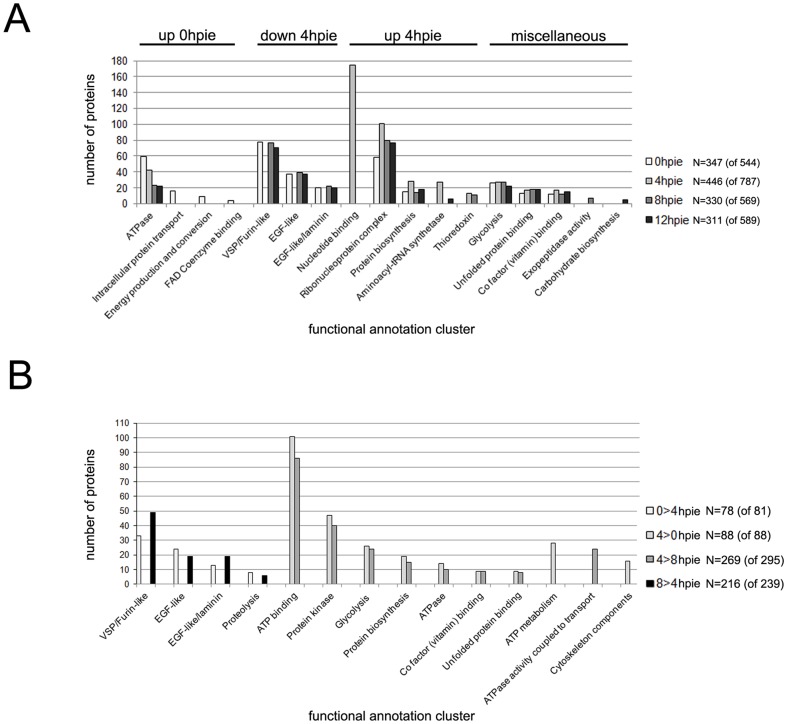
Functional annotation for the proteome of *G. lamblia* trophozoites at 0, 4, 8 and 12hpie. (A) Proteins identified at 0, 4, 8 or 12hpie were assigned to a total of 17 functional annotation clusters using DAVID. Only clusters with enrichment scores of at least 1 were included in the graph and were further discussed. The number of proteins in each cluster dataset is indicated, followed by the total number of proteins submitted to DAVID (in brackets). Functional clusters that were up-regulated at 0hpie (up 0hpie) and that were either up- or down-regulated at 4hpie (up 4hpie and down 4hpie, respectively) are indicated on the graph. hpie: hours post induction of encystation. (B) Significantly regulated proteins between 0 and 4hpie (0>4hpie and 4>0hpie) and 4 and 8hpie (4>8hpie and 8>4hpie) were assigned to functional annotation clusters using DAVID. The number of proteins in each cluster dataset is indicated, followed by the total number of proteins submitted to DAVID (in brackets). Only clusters with enrichment scores of at least 1 were included in the graph and were further discussed. hpie: hours post induction of encystation.

To determine changes in the Giardia metabolic network during encystation, we applied the DAVID algorithm suite [45] for parsing the datasets using functional categories. Following the conversion of the detected *G. lamblia* ORFs to DAVID-compatible identification numbers, we subjected the resulting gene lists to the DAVID web-interface tool for functional annotation clustering, based, amongst other parameters, on available gene ontology (GO) terms, protein-protein interaction data, protein functional domains and bio-pathways. We performed this analysis for all proteins identified at all 4 time-points and detected 17 functional clusters with an enrichment score (ES) of ≥1 (Figure 4A, Table S4). These clusters showed enrichment, depletion or modest changes at 0, 4, 8 and 12hpie (Figure 4A). We performed a similar analysis for all significantly regulated proteins between 0 and 4hpie, and between 4 and 8hpie (Figure 4B, Table S4). Due to the insufficient number of significantly regulated proteins between 8 and 12hpie, we were unable to run a similar analysis.

Four metabolic clusters, “ATPase”, “Intracellular protein transport”, “Energy production and conversion” and “FAD coenzyme binding”, were overrepresented in the 0hpie dataset (Figure 4A, Table S4). The “Intracellular protein transport” cluster contains the clathrin heavy chain and 5 adaptins which were previously shown to localize to peripheral vesicles [46], including 2 vacuolar protein sorting components. Furthermore, the two proteins Sec61 translocon (GL50803_5744) and coatomer β subunit (GL50803_88082) are related to the early secretory pathway. Interestingly, this cluster also includes a hypothetical protein (GL50803_93278) which carries a conserved importin β-related nuclear transport receptor domain (NCBI Conserved domain search, 1.62e^−11^) responsible for nuclear cargo interactions with the nuclear pore complex [47]. We observed that the abundance of proteins involved in intracellular trafficking were generally higher at 4, 8 and 12hpie compared to 0hpie, supporting the earlier observation of a remodeling of intracellular protein transport during encystation (Table S2). In addition, 3 functional clusters associated to surface antigens (“VSP/Furin-like”, EGF-like” and “EGF-like/laminin”) were underrepresented at 4hpie (Figure 4A, Table S4). This is consistent with our observations on the loss of VSP diversity in the early stages of encystation (Table S3). This trend was further highlighted in the functional annotation of proteins significantly more abundant at 0hpie and 8hpie with respect to 4hpie (0>4 and 8>4, Figure 4B and Table S4).

### Several metabolic functions are differentially regulated in early encystation

Many changes in protein abundances took place at 4hpie (Figure 3A). Proteins identified at 4hpie or significantly more abundant at 4hpie belonged to several metabolic functional clusters (Figure 4A and 4B, and Table S4).The functional clusters “Nucleotide binding”, “Ribonucleoprotein complex”, “Protein biosynthesis” and “Aminoacyl tRNA synthetase” were overrepresented at 4hpie (Figure 4A, Table S4), with the first category enriched exclusively at 4hpie (Figure 4A, Table S4). These clusters are likely linked to a general increase in metabolic activity including transcription and translation early during encystation. The “Thioredoxin” cluster is enriched at 4hpie and 8hpie (Figure 4A, Table S4) and it includes 3 protein disulfide isomerases (PDI) and 2 members of the peroxiredoxin (PRX) family. The *G. lamblia* genome has 5 genes coding for PDIs, 3 of which have been localized to the ER [48] and likely aid in correct protein folding. The abundance of the three PDIs identified here increased at 4hpie, suggesting that enhanced protein folding is accompanying increased secretion during encystation. In contrast, identified PRXs were less abundant at 4hpie. PRXs play an important role in relieving cells from oxidative stress caused by hydrogen peroxide [49]. Although it is unlikely that *in vitro* O_2_ concentration would change significantly, *in vivo* encysting parasites may encounter local variations in O_2_ levels while moving towards and through the large intestine [50]. This change in environment may require an adjustment of PRX activity.

Ten metabolic clusters were enriched for proteins significantly more abundant at 4hpie compared to either 0hpie (4>0hpie, Figure 4B) or 8hpie (4>8hpie, Figure 4B). The surge in metabolic regulation at 4hpie is mirrored by an enrichment for protein kinases, several of which were significantly more abundant at 4hpie than at 0 or 8hpie (Figure 4B). Despite Giardia's “reduced kinome” [51], the NIMA-related serine/threonine kinases (NEK) kinase family has seen a massive expansion in this lineage, with up to 71% of all kinase-related ORFs predicted to encode for NEK kinases [51]. Interestingly, *ca.* 70% of these lacks key residues required for substrate phosphorylation, raising the question of their actual catalytic activity [51]. Interestingly, the rhoptry kinase family in *T. gondii* includes several pseudokinases involved in assisting active kinases in exerting their function [52]. It is therefore possible that the majority of NEK kinases in *G. lamblia* may in fact be pseudokinases. The majority (40%, N = 16) of all up-regulated kinases at 4hpie compared to 0hpie were NEK and NEK-like kinases (Table S4). Although regulated less than 2-fold, transcript levels for six of these (ORFs GL50803_ 5489, 17231, 11390, 113553 and 16824) were shown to be higher at 3hpie compared to 0hpie [7]. Further in line with our data, NEK kinase GL50803_15409 was found to be up-regulated early in encystation on both transcript and protein levels [22]. We furthermore identified two kinases, NEK1 and NEK2 (GL50803_92498 and GL50803_5375), that were shown to regulate cell cycle progression, growth and cytokinesis [53]. NEK2 was significantly more abundant at 4hpie while the abundance of NEK1 remained unchanged during encystation (Table S2). The B' regulatory subunit of the highly conserved serine/threonine protein phosphatase 2A (PP2A; GL50803_16443) was also specifically upregulated at 4hpie (Table S2). Importantly, PP2A was previously implicated in *G. lamblia* encystation and was proposed to be also involved in *Trypanosoma cruzi* and *Plasmodium falciparum* differentiation [54].

Several proteins significantly more abundant at 4hpie were involved in glycolysis (Figure 4B). A notable member of this “Glycolysis” cluster is enolase (GL50803_11118) (table S4). This enzyme is unconventionally secreted from trophozoites during colonization and proliferation in the small intestine [55], suggesting a role in Giardia virulence and pathogenicity [56]. An increase in intracellular levels of enolase is consistent with the decrease in unconventional enolase secretion observed in encysting trophozoites [56]. Although not involved in glycolysis, arginine deiminase (ADI; GL50803_112103) is another example of unconventional protein secretion in Giardia trophozoites [55] which is also significantly upregulated at 4hpie compared to all other tested time-points. Aside from enolase, phosphoglycerate kinase (GL50803_90872), pyruvate kinase (GL50803_3206 and GL50803_17143), fructose-bisphosphate aldolase (GL50803_11043), glucokinase (GL50803_8826), glucose-6-phosphate isomerase (GL50803_9115) and pyrophosphate-fructose 6-phosphate 1-phosphotransferase alpha subunit (GL50803_14993) were all consistently more abundant at 4hpie (Table S2), thus demonstrating a distinct regulation of the glycolytic pathway [57].

We observed that all subunits of the chaperonin T-complex 1 (TCP-1) included in the “Unfolded protein binding” cluster were significantly more abundant at 4hpie (Figure 3B and Table S4). The TCP-1 heteromer is essential in yeast and is postulated to be the cytosol-resident eukaryotic equivalent of the prokaryotic groEL system [58]. TCP-1 aids in the ATP-dependent folding of several proteins, including actin and tubulin [59]. Although these data could be interpreted in the context of a stress response to an increased demand for protein folding (see above), our group has recently shown how the regulation of gene expression in encysting trophozoites is clearly distinct from that of cells subjected to stresses which typically induce the unfolded protein response (UPR) [7], [60]. Interestingly, TCP-1 was shown to be necessary for histone deacetylase 3 (HDAC3) activity by promoting HDAC interaction with its nuclear receptor co-repressor SMRT [61]. Giardia presents a single HDAC (GL50803_3281) whose chemical inhibition was shown to inhibit encystation and expression of CWP genes, indicating that de-acetylation plays an important role in stage-conversion [8]. Although giardial HDAC was never detected in our experiment, the higher abundance of TCP-1 components at 4hpie could be linked to increasing HDAC de-acetylating activity which regulates expression of encystation-related genes in the early stages of this process [8].

Importantly, components of the cytoskeleton were specifically enriched at 4hpie with respect to 0hpie (Figure 4B, Table S4). The majority of these significantly regulated proteins were either dyneins or kinesins. These proteins act as molecular motors to mediate ATP-dependent cellular cargo transport along microtubules [62], dyneins towards the minus end of a microtubule (MTOC), kinesins towards the plus end. Both protein types are implicated in organellar transport within the cell including mitochondria, lysosomes and the ER [63]. Aside from suggesting a general re-organization of subcellular structures in early encystation, the higher abundance of motor proteins at 4hpie may be linked to the transport and distribution of newly-formed ESVs which are generally independent of the ER at 5–6hpie [10]. In line with remodeling and internalization of the flagellar apparatus in encysting cells [16], we also identified 14 out of 25 basal body proteins (Tables S1 and S2) whose confident localization was reported in a recent study integrating genomic predictions, proteomics and immuno-localization data [23]. Five of these (GL50803_ 9030, 16745, 16532, 16220, 13766) were also significantly more abundant at 4hpie (Table S2). A striking feature of Giardia encystation is disassembly of the adhesive disk [16]. We detected 22 of 38 previously published adhesive disk candidates [64], [65], 3 of which (GL50803_101291, 103676 and 13584) were at most 2-fold more abundant at 4hpie (Table S2). This is consistent with previous reports of 2D PAGE analyses showing little change in adhesive disk protein abundance during encystation [16]. Furthermore, we detected 7 putative giardial ankyrins containing coiled-coil domains which were localized to flagellar elements and have been proposed to play a role in flagellar activity [66]. In mammalian cells, ankyrins were identified as a family of adaptor proteins that mediate the attachment of integral membrane proteins to the spectrin-actin based membrane cytoskeleton [67]. Although ankyrin function in the giardial cell awaits a more detailed characterization, specific induction of this protein family in the early stages of encystation may serve as a novel marker for this process. Overall, our data indicates how cells in early encystation undergo significant metabolic changes which include (sub)cellular structure remodeling.

### The Giardia proteome undergoes only modest changes in the later stages of encysation

Only few functional clusters were enriched at later stages of encystation (Figures 4A and 4B, Table S4). Notably, the 8hpie dataset presented an exclusive enrichment for the “Exopeptidase activity” cluster which includes 2 metallo-proteases of the insulinase family (GL50803_93551 and GL50803_9508). Consistent with their significantly increased protein abundances at 4hpie, transcripts for these 2 metallo-proteases were previously found to be positively regulated during encystation, generally at 3hpie [7]. Proteins involved in “Proteolysis” were also found to be enriched at 0hpie and 8hpie compared to 4hpie (Figure 4B, Table S4). Interestingly, the giardial serpin 1 homologue (ORF GL50803_4653) was also significantly more abundant at 0 and 8hpie with respect to 4hpie. Serpins constitute a superfamily of irreversible serine protease inhibitors, present in all living organisms and in Poxviruses [68]. A decrease in serpin-dependent inhibitory activity in the early stages of encystation could promote protein turnover by serine proteases, thereby contributing to the metabolic and cellular remodeling of early encysting trophozoites. Further experimental data is therefore required to determine whether an important aspect of *G. lamblia* encystation is perhaps regulation of serine protease activity [69]. In line with this hypothesis, 1 of only 7 significantly up-regulated proteins at 12hpie with respect to 8hpie was a putative furin precursor serine protease (GL50803_2897), also referred to as SPC for subtilisin-like preprotein convertase [70]. Our data is in agreement with previous reports on the overexpression of both the protein and the corresponding transcript in late encystation [7], [70]. The canonical catalytic triad for this class of peptidases was not found in the giardial homologue and its substrate(s) remain uncharacterized [70]. Further work is therefore required to establish whether this “atypical” preprotein convertase may function as a pseudo-enzyme involved in the regulation of specific proteolytic events occurring in the later stages of encystation [71].

Our last time-point at 12hpie presented just 1 enriched cluster for “Carbohydrate biosynthesis” (Figure 4A). Interestingly, only one protein in this cluster, glucose-6-phosphate isomerase (GL50803_9115), has so far been implicated in encystation-dependent GalNAc biosynthesis [30] (Table S4). Other cluster members include a putative glycogen synthase (GL50803_104031) and the glycogen debranching enzyme 4-alpha-glucanotransferase-amylo-alpha-1,6-glucosidase (GL50803_10885), although a role for glycogen metabolism in the later stages of encystation has not been described so far.

An interesting observation was the significant increase of giardial H2A histone (GL50803_14256) at 12hpie. The entire core histone repertoire in *G. lamblia* is composed of histones H2A and H2B, H3 and H4 [72]. No H1 histone-encoding ORF has yet been identified in the giardial genome sequence, suggesting that condensation of DNA in this organism may involve non-conventional nucleosome assembly [73]. The overexpression of H2A in the later stages of encystation could be explained with ensuing rounds of DNA replication required to obtain a tetranucleate 16N cyst from a binucleate 4N trophozoite, although we did not detect a parallel increase in the protein levels for other core histones. In line with our data, transcript levels for H2A histone did not change significantly in the earlier stages of encystation and at 7hpie [7], suggesting that the overexpression we observed is indeed limited to the later stages of encystation. None of the transcripts for the other core histones were regulated more than 2 fold during encystation [7]. SAGE data obtained using an alternative encystation protocol showed less than 2-fold increase of H2B expression during encystation (GL50803_121045, GL50803_121046) while the abundance of H3 mRNA (GL50803_3367, GL50803_14212 and GL50803_135231) remained unchanged or very slightly decreased [19]. Interestingly, the H2A-encoding mRNA was found to be a target of miRNA 3 ([74]; user comment by A. Saraiya on www.giardiadb.org). There is currently no information on the regulation of miRNA 3 during encystation, thus the biological interpretation and significance of this observation awaits further investigation.

## Conclusion

In this study, we present the first large-scale quantitative analysis of the *G. lamblia* proteome and it's regulation during *in vitro* encystation. Widespread changes in protein abundance were detected in the early stages of this differentiation process which was marked by a remodeling of the cell's surface through the regulation of VSPs. Our findings raise important questions regarding the role and regulation of antigenic switching in encysting populations. In parallel, enrichment for motor proteins such as dyneins and kinesins suggest a re-arrangement of subcellular compartments prior to ESV neogenesis. Importantly, our data confirms and expands several previous reports identifying encystation markers and provides broad insight into novel protein targets regulated during encystation.

Previous work reported on the absence of sweeping changes in gene expression during encystation [7], [19], leading to the hypothesis that regulation in protein abundance may have followed a similar trend. On the contrary, our data demonstrates differential regulation of several hundred proteins, suggesting an important role for post-transcriptional control of gene expression during parasite differentiation. A case in point is EGFCP1, a non-CWP ESV component whose protein levels were shown to increase during encystation, with no significant changes in corresponding transcript abundance [75].

The wealth of presented data awaits further hypothesis-driven mining, experimental validation and functional analysis. This is all the more relevant when we consider that currently almost 70% of the entire predicted proteome for *G. lamblia* WB assemblage A is annotated as “hypothetical”. Improving annotation for the *G. lamblia* proteome will serve many purposes, including earmarking of specific metabolic processes which may be also regulated during ERIF formation in other parasitic protozoan species [76], [77]. The presented data has been submitted for integration in the GGD platform and we believe that its release to the scientific community will contribute to the further understanding of the striking process of encystation, which is key to parasite survival based on alternating between the trophozoite and the cyst stage.

## Materials and Methods

### Giardia cell culture and induction of in vitro encystation

Trophozoites of the *G. lamblia* strain WBC6 (ATCC catalog number 50803) were grown under microaerophilic conditions in 11 ml culture tubes (Nunc, Roskilde, Denmark) containing TYI-S-33 medium supplemented with 10% adult bovine serum and bovine bile according to standard protocols [27]. Encystation was induced using the 2-step method as described previously [27]. Briefly, cells were cultured for 44 hours to confluence in medium without bile (pre-encysting medium) and subsequently in pre-warmed encystation medium containing porcine bile (0.25 mg/ml; Sigma) and lactic acid (0.545 mg/ml; Sigma) at pH 7.85. 40 million attached cells were harvested at given hours post induction of encystation (hpie); cell debris, cysts and spontaneously-encysting trophozoites were discarded with the medium.

### Immuno-fluorescence analysis

Chemical fixation and preparation for fluorescence microscopy was performed as described [14]. Briefly, cells were washed with cold PBS after harvesting and fixed with 3% formaldehyde in PBS for 40 min at 20°C, followed by 5 minutes incubation with 0.1 M glycine in PBS. Cells were permeabilized with 0.2% triton X-100 in PBS for 20 min at room temperature and blocked overnight in 2% BSA in PBS. Incubations of all antibodies were done in 2% BSA/0.2% Triton X-100 in PBS for 1 h at 4°C. The following antibodies were used in this work: Texas Red-conjugated anti-CWP1 (Waterborne™, Inc., New Orleans, LA, USA; dilution 1∶80). Post incubation washes were done with 1% BSA/0.1% triton X-100 in PBS. Labeled cells were embedded for microscopy with Vectashield (Vector Laboratories, Inc., Burlingame, CA, USA) containing the DNA intercalating agent 4′-6-Diamidino-2-phenylindole (DAPI) for detection of nuclear DNA. Immuno-fluorescence analysis was performed on a Leica DM IRBE microscope (Leica Microsystems, Wetzlar, Germany) equipped with an oil immersion objective (Leica, HCX PL FLUOTAR PH3 100X).

### Protein extraction and mass-spectrometry based protein identification

Protein extracts were prepared by lysing shock-frozen Giardia cells in 0.3 ml of a Tris-based SDS buffer (40 mM Tris, pH 8.0, 40 mM DTT, 5 mM MgCl_2_, 4% SDS, and 1× protease inhibitor cocktail [Calbiochem]). Cell lysates were clarified by centrifuging twice at 20,000 rcf for 20 min at room temperature; the supernatant fraction only was retained for further processing. Protein concentration was determined using a BCA protein assay kit (Thermo Scientific-Pierce) before adding 40 mM DTT and similar protein concentrations were obtained for all time points (∼10 µg/µl). 40 micrograms of total protein for each time-point were subjected to SDS-PAGE on 12% resolving gels. After electrophoresis, gels were stained with Coomassie Brilliant Blue according to standard procedures and each sample lane was cut into 10 sections. Each gel slice was then diced into smaller pieces. In-gel digestion was performed according to previous reports [78]. After digestion, dried peptides were resuspended in 3% acetonitrile and 0.2% trifluoretic acid and desalted using Sepak cartridges (Waters).

Dried peptides were resuspended in 3% acetonitrile and 0.2% formic acid and analyzed on a LTQ Orbitrap Discovery mass spectrometer (Thermo Fischer Scientific, Bremen, Germany) coupled to an Eksigent-Nano-HPLC system (Eksigent Technologies, Dublin (CA), USA). Peptide mixtures were loaded onto laboratory-made capillary columns (75 µm inner diameter (BGB Analytik, Böckten, Switzerland), 8 cm length, packed with Magic C18 AQ beads, 3 µm, 100 Å (Michrom BioResources, Auburn, CA, USA)). Peptides were eluted from the column by an increased acetonitrile concentration in the mobile phase from 5% (v/v) acetonitrile, 0.2% (v/v) formic acid to 40% (v/v) acetonitrile, 0.2% (v/v) formic acid over 74 minutes, followed by a 10 minutes wash step at 5% (v/v) acetonitrile, 0.2% (v/v) formic acid at a flow rate of 200 nl/minute. Full-scan MS spectra (300–2000 m/z) were acquired with a resolution of 60000 at 400 m/z after accumulation to a target value of 500000. Collision induced dissociation (CID) MS/MS spectra were recorded in data dependent manner in the ion trap from the six most intense signals above a threshold of 500, using a normalized collision energy of 35% and an activation time of 30 ms. Charge state screening was enabled and singly charge states were rejected. Precursor masses already selected for MS/MS were excluded for further selection for 120 s and the exclusion window was set to 20 ppm. The size of the exclusion list was set to a maximum of 500 entries. The spectral output of all measured samples was stored in a .raw file format from the software Xcalibur used on the Thermo Fischer mass spectrometers (Thermo Fischer Scientific, Bremen, Germany). These files were subsequently transformed in a .mgf format using the Mascot software suite (Matrix Science), allowing for database searches and protein identification.

To identify measured peptides, we generated a proteome database using the Giardia Genome release-1.2 (25-Jun-2009). We used the file “GlambliaAnnotatedProteins_GiardiaDB-1.2.fasta” (17-Aug-2009) corresponding to the proteins, translated CDS (AA). To the forward protein sequences, we added the respective reverse sequences to be able to determine the false positive rate of protein and peptide discovery. To allow for correct peptide and protein identification we increased the size of the Giardia proteomics database by adding protein sequences from *Arabidopsis thaliana* (TAIR9). MS/MS spectra were searched with Mascot version 2.2.04 against this database with a concatenated decoy database supplemented with contaminants (67,079 entries). The search parameters were as follows: requirement for semitryptic ends, one missed cleavage allowed, mass tolerance = ±5 ppm. Besides carbamidomethylation of Cys residues as fixed modification, oxidation of Met was included as a variable modification. Identified peptides were accepted with a Mascot ion score higher than 23 and a Mascot expectation value smaller than 0.05. To increase protein identification confidence, a minimum of two unique peptides for each identified protein was required. The spectrum false discovery rate was calculated by dividing the number of decoy database spectrum assignments by the number of spectrum assignments in the final dataset. The false positive rate was below 1% for all measured biological replicates.

Protein quantification with nSpC was done according to [25] and [26]. Briefly, the expected contribution of each individual protein to the samples total peptide pool was calculated correcting the values with a normalization factor, which balances for the theoretical number of tryptic peptides per protein and sample depth according to the following formula: nSpC_K_ = Spectra_K_×((TTP_K_×MS)/MP)^−1^ where nSpCK is the normalized spectral count for protein K, TTPK is the theoretical tryptic peptides of protein K, MS is the total number of measured spectra in the dataset, and MP is the total number of theoretical tryptic peptides of the identified proteins in the dataset.

For the determination of the number of TTPK, the whole protein database was digested in silico according to Baerenfaller et al., 2008. If Arg or Lys was followed by Pro (KP/RP site), the site was both cut and not cut (resulting in 3 tryptic peptides). If several of these sequence pairs followed each other, we only considered cutting of one KP/RP site per time. Resulting peptides were labeled as theoretical tryptic peptides, which were between 400 and 6000 Da, at least 6 amino acids long, and contained a tryptic start and end.

### Access to proteomics data on PRIDE

The measured data was exported to the PRIDE database (www.ebi.ac.uk/pride; [33]) (login: review15038; password: j∧jHZtPD). Accession numbers for the 14 hour time-course are 26860, 26861, 26866, 26868, 26869–71 and 26878. Accession numbers for the in-depth analysis with biological triplicates are 26862–5, 26867, 26872–7 and 26879.

### Web-based protein localization and functional prediction tools

Protein hits in FASTA format were uploaded to the web-interface of prediction algorithm Target P [37] at http://www.cbs.dtu.dk/services/TargetP. Based on the indications on the algorithm's web-interface, reliability class 3 was set as the threshold for statistically-significant *in silico* predictions, with RC 1 associated to the most robust predictions. Protein hits were further analyzed for putative nuclear localization signals using the web-based prediction algorithm NucPred [38] at http://www.sbc.su.se/~maccallr/nucpred/. Based on the indications on the algorithm's web-interface, the prediction reliability score cut-off was set to 0.7; nuclear localization predictions with a score of 0.7 and above were considered significant. The DAVID algorithm suite [45] was used to detect specific functional clusters within the protein datasets. Protein hits identified by giardial ORF codes such as GL50803_XXXXX were copied to a .txt file and converted to gene identification numbers (Gene IDs) by uploading the corresponding .txt file and selecting “Gene” from the drop-down menu on the NCBI Batch Entrez tool at http://www.ncbi.nlm.nih.gov/sites/batchentrez. Not all *G. lamblia* ORFs could be assigned a Gene ID number however, resulting in a reduction of the original datasets to a shorter gene list. The resulting Gene IDs were exported to a new file and pasted as gene lists on the DAVID web-interface tool for functional annotation clustering at http://david.abcc.ncifcrf.gov/summary.jsp. Following the conversion of this gene list to DAVID identification numbers, the functional clustering analysis was launched. When sufficient data is available, the functional annotation clustering tool groups and displays contents from the same or different resources into annotation groups. Each group was then assigned an enrichment score (ES) which indicates the prominence of a specific functional group within a given list of genes. Only clusters with a ES≥1 were selected for further analysis [45].

## Supporting Information

Figure S1 (A) 40 µg of total protein was extracted from *G. lamblia* trophozoites sampled at 0, 2, 4, 6, 8, 10, 12 and 14hpie. Following resolution by one-dimension SDS-PAGE, each gel lane was cut to 10 pieces which were then separately subjected to in-gel tryptic digestion. Resulting peptides were measured by tandem mass spectrometry. Approximate protein molecular weights in kDa are indicated on the left. hpie: hours post induction of encystation. (B) Protein data overview across the 14 hour time-course experiment, including the overall number of identified proteins for each time-point and the number of proteins in common across time-points. hpie: hours post induction of encystation. (C) Distribution box-plots for the comparison of label-free proteomics data at 0hpie from this study to RNA-seq data reported in Franzén et al., 2013 [21]. Protein abundance in the upper box-plot is expressed using normalized spectral counting (nSpC) while RNA abundance in the lower box-plot is expressed as fragments per kilobase per million fragments mapped (FPKM); in brackets, the overall number of proteins for each dataset is indicated. Lower and upper quartiles are shaded in dark and light grey, respectively. Both datasets show how protein and transcript products derived from annotated ORFs are more abundant than products of hypothetical ORFs.
(TIF)

Figure S2 **Distribution box-plots comparing the abundance across time-points of the 100 most abundant proteins identified at 0hpie (A), 4hpie (B), 8hpie (C) and 12hpie (D).** Protein abundance is represented using normalized spectral counting (nSpC) while lower and upper quartiles are shaded in dark and light grey, respectively. The plots show how the abundance of highly abundant proteins at 0hpie is reduced at 4hpie.
(TIF)

Table S1 **All proteins identified at 0, 2, 4, 6, 8, 10, 12 and 14 hours post induction of encystation.** The normalized spectral counting (nSpC) and the total number of identified peptides are given. Differentially abundant proteins between two time-points were required to have a minimum twofold change in abundance (Ratio nSpC) and to have been identified with at least 5 peptides in one of the two time-points. Proteins only detected in one time point were also required to have a number of at least 5 total peptides to be considered significantly more abundant.
(XLSX)

Table S2 **All proteins identified at 0, 4, 8 and 12 hours post induction of encystation.** The normalized spectral counting (nSpC) and the total number of identified peptides (TniP) are given. The T-test was performed on the nSpC of three biological replicates. Differentially abundant proteins between two time-points were required to have a minimum twofold change in abundance (Ratio mean nSpC) and to have been identified with at least 5 peptides in one of the two time-points. Proteins detected in just one time point were also required to have at least 5 peptides to be considered significantly more abundant.
(XLSX)

Table S3 **All 47 variant-specific surface proteins (VSPs) proteins identified at 0, 4, 8 and 12 hours post induction of encystation.** The normalized spectral counting (nSpC) and the total number of identified peptides (TniP) are given. The T-test was performed on the nSpC of three biological replicates. Differentially abundant proteins between two time-points were required to have a minimum twofold change in abundance (Ratio mean nSpC) and to have been identified with at least of 5 peptides in one of the two time-points. Proteins only detected in one time point were also required to have at least 5 peptides to be considered significantly more abundant.
(XLSX)

Table S4 **Functional annotation clustering analysis performed with DAVID for each time-point and for all significantly regulated proteins between 0 and 4hpie (0>4hpie and 4>0hpie) and 4 and 8hpie (4>8hpie and 8>4hpie).** Only clusters with enrichment scores (ES) of at least 1 were considered for further discussion and included in this table. Each entry in the table contains multiple ORF numbers assigned to each cluster. hpie: hours post induction of encystation.
(XLSX)
