# Supplementary figures and images for: The Proteome Landscape of Giardia lamblia Encystation
Source: PLoS One. 2013 Dec 31;8(12):e83207. doi: 10.1371/journal.pone.0083207 (PMC3877021; doi:10.1371/journal.pone.0083207)

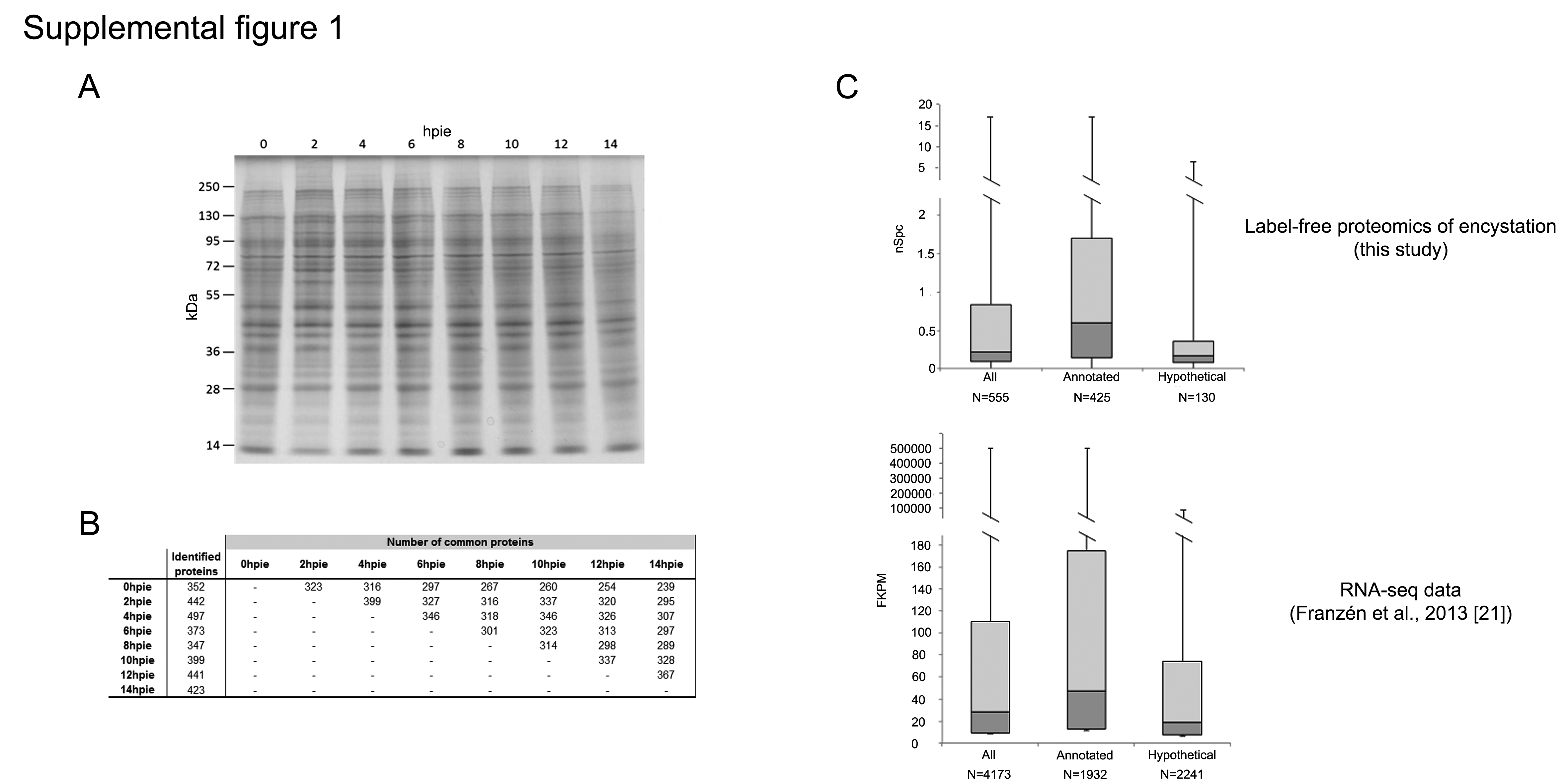

Supplement: Figure S1 — (A) 40 µg of total protein was extracted from G. lamblia trophozoites sampled at 0, 2, 4, 6, 8, 10, 12 and 14hpie. Following resolution by one-dimension SDS-PAGE, each gel lane was cut to 10 pieces which were then separately subjected to in-gel tryptic digestion. Resulting peptides were measured by tandem mass spectrometry. Approximate protein molecular weights in kDa are indicated on the left. hpie: hours post induction of encystation. (B) Protein data overview across the 14 hour time-course experiment, including the overall number of identified proteins for each time-point and the number of proteins in common across time-points. hpie: hours post induction of encystation. (C) Distribution box-plots for the comparison of label-free proteomics data at 0hpie from this study to RNA-seq data reported in Franzén et al., 2013 [21]. Protein abundance in the upper box-plot is expressed using normalized spectral counting (nSpC) while RNA abundance in the lower box-plot is expressed as fragments per kilobase per million fragments mapped (FPKM); in brackets, the overall number of proteins for each dataset is indicated. Lower and upper quartiles are shaded in dark and light grey, respectively. Both datasets show how protein and transcript products derived from annotated ORFs are more abundant than products of hypothetical ORFs. (TIF) [file pone.0083207.s001.tif]

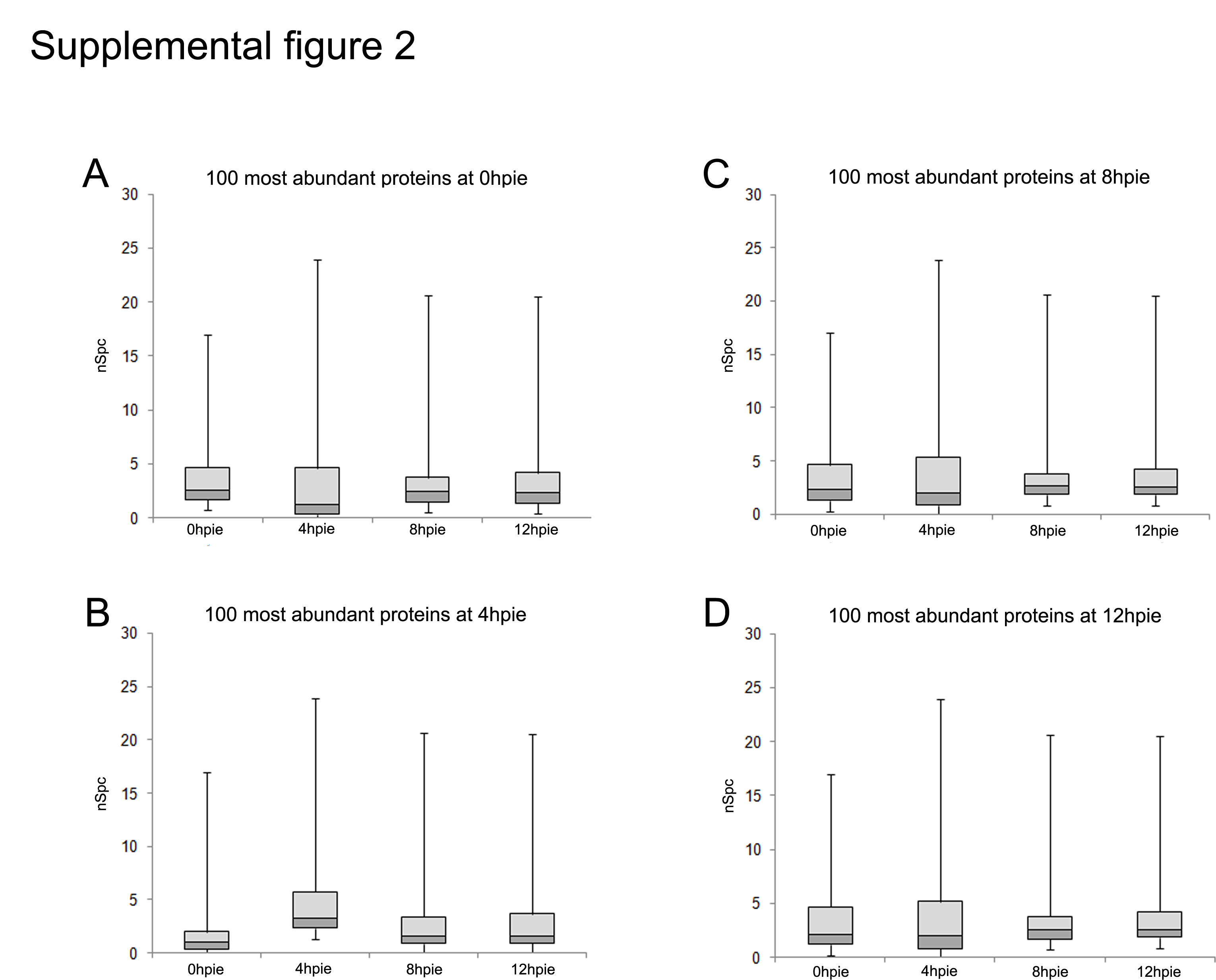

Supplement: Figure S2 — Distribution box-plots comparing the abundance across time-points of the 100 most abundant proteins identified at 0hpie (A), 4hpie (B), 8hpie (C) and 12hpie (D). Protein abundance is represented using normalized spectral counting (nSpC) while lower and upper quartiles are shaded in dark and light grey, respectively. The plots show how the abundance of highly abundant proteins at 0hpie is reduced at 4hpie. (TIF) [file pone.0083207.s002.tif]
